# Correction: Esmaeilzadeh et al. RETRACTED: Identify Biomarkers and Design Effective Multi-Target Drugs in Ovarian Cancer: Hit Network-Target Sets Model Optimizing. *Biology* 2022, *11,* 1851

**DOI:** 10.3390/biology13090666

**Published:** 2024-08-27

**Authors:** Biology Editorial Office

**Affiliations:** MDPI, St. Alban-Anlage 66, 4052 Basel, Switzerland; biology@mdpi.com

A correction has been made to the authorship list of this article [1] and the linked retraction notice [2]. Following the publication of the above-mentioned retraction, the Editorial Office was made aware that certain individuals previously listed as authors were unaware of the submission and did not contribute to the article. As a result, the authorship list of the article [1] and the retraction notice [2] have been updated accordingly. This correction was approved by the Editors-in-Chief. This correction does not affect the content of the retraction notice.
